# Neuroendocrine tumour in a patient with neurofibromatosis type 1 and HIV

**DOI:** 10.4102/sajhivmed.v16i1.323

**Published:** 2015-06-26

**Authors:** Juliane Hiesgen, Ebrahim Variava

**Affiliations:** 1 Department of Neurology, Kalafong Hospital, University of Pretoria, South Africa; 2 Department of Internal Medicine, Tshepong Hospital, University of the Witwatersrand, South Africa

## Abstract

We report the case of an HIV-positive female patient with neurofibromatosis type 1 who was treated for recurrent peptic ulcer disease and later developed diabetes mellitus and chronic diarrhoea. A metastasising somatostatinoma was histologically proven and evidence of a concomitant gastrin-producing neuroendocrine tumour was found. Neuroendocrine tumours (NETs) are very rare neoplasms originating from a wide variety of endocrine and nervous system tissue with the ability to produce different hormones. A somatostatin- and gastrin-secreting NET in a patient with HIV has not been reported in the literature, to the best of our knowledge. We discuss oncogenic pathomechanisms related to the underlying conditions and propose stringent monitoring for tumours in HIV-positive patients with phakomatoses as well as initiation of antiretroviral therapy.

## Introduction

Neuroendocrine tumours (NETs) are neoplasms originating from a wide variety of endocrine and nervous system tissues with the ability to produce different hormones. Pancreatic hormone-producing NETs are often associated with specific clinical manifestations, resulting from the excessive production and action of the respective hormone.

We report on a 30-year-old HIV-positive female patient with neurofibromatosis type 1 (NF1) who presented with Zollinger-Ellison syndrome and later developed diabetes mellitus and chronic diarrhoea. A somatostatinoma was proven histologically and we found laboratory evidence for the concomitant production of gastrin by the tumour. Somatostatinomas are very rare tumours, and a somatostatin- and gastrin-secreting NET in a patient with HIV has not been reported. We discuss oncogenic pathomechanisms related to the underlying conditions and propose stringent monitoring for tumours in HIV-positive patients with phakomatoses.

## Case presentation

A 30-year-old woman had been seen at the Surgical Department over the past five years for recurrent upper gastro-intestinal bleeds. Peptic ulcer disease was proven gastroscopically on four occasions. She was repeatedly treated with proton-pump inhibitors and on one occasion received empiric triple therapy for *Helicobacter pylori* eradication. Her past medical history included visits to the Plastic Surgery Department for removal of a plexiform neurofibroma with enucleation of her right eye.

Clinically, with numerous neurofibromata, one big plexiform neurofibroma on the right side of her face, several café-au-lait spots and axillary freckling, she fulfilled the criteria for NF1.^1^ She had tested HIV-positive a few months earlier, with a baseline CD4 count of 290 cells/µL. One week prior to admission, diabetes mellitus was diagnosed by the local primary healthcare clinic and she was started on metformin 850 mg bd. She then presented to the Medical Department with a four-day history of diarrhoea without melaena or epigastric pain.

**FIGURE 1:**
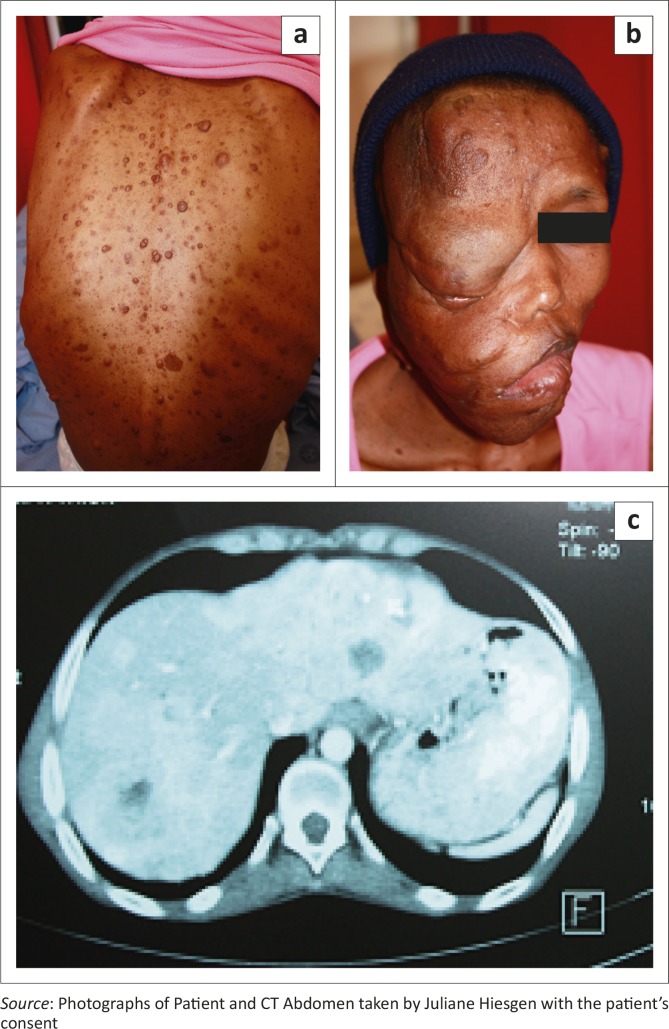
Clinical features of NF 1, with numerous neurofibromata (a), big plexiform neurofibroma over the right side of the face (b) and (c) CT image showing several hypodense lesions in the liver parenchyma, suggestive of metastases. *Source*: Photographs of Patient and CT Abdomen taken by Juliane Hiesgen with the patient's consent

On admission, she was moderately dehydrated with features of NF1 (Figures 1a and 1b). The rest of the clinical examination was normal. A chest X-ray was unremarkable, and blood results were essentially normal except for a thrombocytosis of 614 x 10^9^ cells/L. The patient received intravenous fluids and antibiotics. Insulin was commenced and antiretroviral therapy (ART) with TDF, 3TC and EFV was started.

**FIGURE 2:**
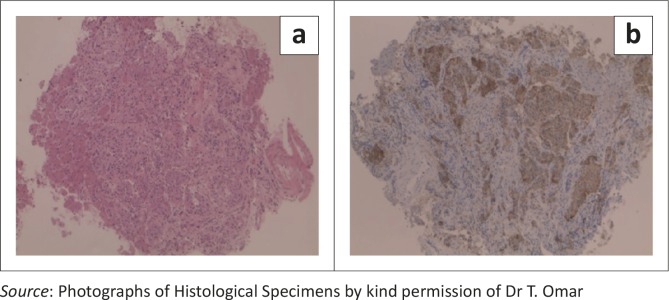
Histological slides from liver lesions: (a) HE staining showing polyglandular tumour cells with granular eosinophilic cytoplasm and glandular formation and (b) strongly positive immuno-histochemical synaptophysin stain (specific for neuroendocrine tumours).

Abdominal ultrasound demonstrated several solid, round lesions in the liver that were confirmed on computed tomography (Figure 1c). Liver biopsy showed polygonal tumour cells with granular eosinophilic cytoplasm and monomorphic nuclei, coarse dispersed chromatin and focal glandular formations. Synaptophysin and chromogranin stains were positive, compatible with a metastatic neuroendocrine carcinoma. Special somatostatin staining was positive, proving the somatostatinoma (Figure 2a and 2b). The excessively increased fasting gastrin level of 19 577 ng/L (13 ng/L – 115 ng/L) strongly suggested the additional hypersecretion of gastrin by the tumour.

A malignant, metastatic neuroendocrine tumour secreting somatostatin and gastrin was diagnosed. Unfortunately, the patient died soon after diagnosis.

## Discussion

Somatostatinomas are extremely rare neuroendocrine tumours with an estimated annual incidence of 1 in 40 million per year, arising from the delta cells in the pancreas or, in about 40% of cases, from the duodenum.^2^ Gender distribution is equal and mean age at presentation is between 51 and 53 years. Occasionally, additional hormones such as glucagon, calcitonin, insulin, gastrin or others are produced. Somatostatinomas often metastasise early and present late, resulting in a poorer prognosis.^2^ Whilst non-metastatic somatostatinomas with full tumour resection can be cured, metastatic forms often have a fatal course as they are diagnosed late. Appropriate surgery combined with chemotherapy results in an average 5-year survival of about 59.9%.^2^

The association between NF1 and somatostatinomas is well documented. Fendrich et al. reported a case of NF1 and a duodenal somatostatinoma, and found 36 other cases in the literature until 2004, of which only 14 involved metastasis.^3^ Somatostatinomas in NF1 occur with a higher frequency and are located more often in the duodenum than in patients without NF1, in whom pancreatic tumours dominate. NETs located in the duodenum tend to present less often with a somatostatinoma syndrome but rather with local or non-specific symptoms.^3^

Most somatostatinomas are symptomatic. The full clinical picture of the somatostatinoma syndrome, characterised as an inhibitory syndrome, was initially reported by Krejs et al. in 1979.^4^ It comprises diabetes mellitus (suppression of insulin), steatorrhoea and cholelithiasis (inhibition of cholecystokinin and biliary motility). Additionally, patients often have general symptoms such as nausea and vomiting, abdominal pain and weight loss. Duodenal somatostatinomas might present with abdominal pain, duodenal obstruction, gastro-intestinal bleeding or jaundice, owing to local growth of the tumour.^2^

The differential diagnosis is wide, depending on the patient's presentation. It includes, amongst others, refractory diabetes mellitus and other endocrine conditions, carcinoids and gastro-intestinal malignancies, pancreatitis, inflammatory bowel disease, coeliac disease, irritable bowel syndrome or even depression.

NF1, originally described by Friedrich von Recklinghausen in 1882, is a fairly common hereditary disease that is autosomal-dominantly inherited and occurs in 1:3500 births. It forms part of the neuro-cutaneous syndromes or phakomatoses, a group of genetic conditions predominantly involving tissues of ectodermal origin, mainly the nervous system, skin and eye. NF1 is characterised by the slow evolution of tumour lesions in childhood and adolescence, as well as by a tendency to form hamartomas and a disposition to fatal malignant transformation. The mutated gene, encoding the protein neurofibromin (17q11.2), is a tumour-suppressor gene.

Involved mechanisms are rat sarcoma viral oncogene homologue (RAS)-mitogen activated protein kinase (MAPK), mammalian target of rapamycin (mTOR) and P21 protein (Cdc42/Rac)-activated kinase (PAK1).^5^ Patients with this disorder are predisposed to both benign and malignant tumours of neurogenic and non-neurogenic origin. NF1 reduces average life expectancy by 10–15 years, with malignant tumours being the most common cause of death.

In addition to NF1, our patient was HIV-positive with a low CD4 count. HIV infection is strongly associated with specific malignancies. Kaposi's sarcoma (KS), non-Hodgkin lymphomas (NHLs) and invasive cervical cancer are AIDS-defining illnesses.^6^

Several non-AIDS-defining malignancies appear more common amongst HIV-positive patients, and their incidence is increasing; these include invasive anal carcinoma, Hodgkin lymphomas, skin cancers, leukaemia, lung cancer, multiple myeloma, prostate cancer and others.^7,8,9,10,11^

Different pathomechanisms, direct and indirect, are involved in the oncogenesis in HIV. Opportunistic co-infections with oncogenic viruses can result in HIV-associated malignancies. Here the role of viral encoded micro-RNA is under investigation.^12^ In particular, the associations of Kaposi's sarcoma and primary effusion NHL with human herpes virus type 8 (HHV 8), of primary CNS lymphoma and NHL with Epstein-Barr virus (EBV) and of invasive cervical cancer with human papillomavirus (HPV) are well documented.^13,14,15^

Immune deficiency itself seems to play a role in HIV malignancies.^16^ Impaired T-cell surveillance in particular may lead to insufficient elimination of transformed cells, resulting in oncogenesis. Furthermore, the HIV TAT protein, a nonstructural protein secreted by infected cells and taken up by uninfected cells, seems to be involved in the pathomechanism of HIV-related malignancies. It has been found to deregulate cellular genes (as pRb2/p130) that work as onco-suppressor proteins.^17,18^

Recently, hyperactivation of mTOR has been found to play a role in different aspects of HIV pathology including HIV-associated nephropathy (HIVAN), HIV encephalopathy, and HIV-associated and non-HIV-associated malignancies.^19,20^ As mentioned above, the mTOR pathway disinhibition is also one of the pathomechanisms involved in the oncogenesis in NF1. Additional mTOR activation in the setting of HIV infection as a compounding contributor may confer a 'second hit’, leading to the question of the potential use of mTOR inhibitors in the treatment of these patients. This point also raises the interesting question of whether HIV patients with an increased risk for malignancies develop these at earlier ages and whether these tumours are more aggressive.

## Conclusion

People living with HIV and/or AIDS have a significantly increased risk of developing malignancies, as have patients with neurofibromatosis (and other phakomatoses). Different mechanisms are involved in these two independent pro-oncogenic diseases, and there are no data on incidence or prevalence rates for patients affected by both conditions. We assume that these rates might be higher than for HIV or NF1 alone.

Therefore, in a setting of high HIV prevalence – such as South Africa – we suggest regular HIV testing in patients with NF1 and other phakomatoses. Frequent follow-up (e.g. 6-monthly) with close monitoring for malignancies and further diagnostic work-up, where a tumour is suspected, is encouraged. Because the risk resulting from the genetic condition is not modifiable, the aim can only be to reduce the tumour risk from HIV infection and immunosuppression. We therefore recommend starting ARVs irrespective of CD4 counts in such patients.
